# Anticancer Potential and Safety Profile of β-Lapachone In Vitro

**DOI:** 10.3390/molecules29061395

**Published:** 2024-03-21

**Authors:** Karina Motta Melo Lima, Luana França Calandrini de Azevedo, Jorge Dores Rissino, Valdicley Vieira Vale, Erica Vanessa Souza Costa, Maria Fani Dolabela, Cleusa Yoshiko Nagamachi, Julio Cesar Pieczarka

**Affiliations:** 1 Center for Advanced Studies of the Biodiversity, Guamá Science and Technology Park, Federal University of Pará, Belém 66075-750, PA, Brazil; luanacalandrini@yahoo.com.br (L.F.C.d.A.); jorgerissino@yahoo.com.br (J.D.R.); cleusanagamachi@gmail.com (C.Y.N.); juliopieczarka@gmail.com (J.C.P.); 2 Campus Tomé Açu, Federal Rural University of the Amazon, Tomé Açu 68680-000, PA, Brazil; 3 Postgraduate Program in Pharmaceutical Innovation, Federal University of Pará, Belém 66075-110, PA, Brazil; valdicleyvale@gmail.com (V.V.V.); rcericavanessa@hotmail.com (E.V.S.C.); fanidolabela@gmail.com (M.F.D.)

**Keywords:** Ipê, antiproliferative activity, genotoxicity, mutagenicity, apoptosis

## Abstract

Ipê is a plant of the Bignoniaceae family. Among the compounds extracted from this tree, lapachol is notable because its structural modification allows the production of β-lapachone, which has anticancer properties. The objective of this work was to test this hypothesis at a cellular level in vitro and assess its potential safety for use. The following tests were performed: MTT cell viability assay, apoptotic index determination, comet assay, and micronucleus test. The results showed that β-lapachone had a high cytotoxic capacity for all cell lines tested: ACP02 (gastric adenocarcinoma cells), MCF7 (breast carcinoma cells), HCT116 (colon cancer cells) and HEPG2 (hepatocellular carcinoma cells). Regarding genotoxicity, the exposure of cells to sublethal doses of β-lapachone induced DNA damage (assessed by the comet assay) and nuclear abnormalities, such as nucleoplasmic bridges and nuclear buds (assessed by the micronucleus test). All tested cell lines responded similarly to β-lapachone, except for ACP02 cells, which were relatively resistant to the cytotoxic effects of the compound in the MTT test. Our results collectively indicate that although β-lapachone showed antiproliferative activity against cancer cell lines, it also caused harmful effects in these cells, suggesting that the use of β-lapachone in treating cancer should be carried out with caution.

## 1. Introduction

Plant-derived agents are important sources of drug constituents and have notably contributed to modern medicine. The bioactive compounds of plants comprise various phytochemicals arising from secondary metabolism; they show therapeutic actions in humans and can be refined to produce drugs [1,2,3].

Ipê is a plant of the Bignoniaceae family. It is native to tropical forests, and infusions obtained from the species have been used medicinally for centuries. The active components of Ipê extract include large quantities of lapachol, which can be structurally altered to the desirable compound, naphthoquinone (β-lapachone) [4,5].

β-Lapachone has been widely studied for its pharmacological effects. Studies have found that it exhibits cytotoxic potential in different types of cancer, including breast cancer [6], prostate cancer [7], multiple myeloma [8,9], hepatocellular carcinoma [10], pancreatic cancer [11,12], human leukemia [13], and others. When studying compounds with potential anticancer activity and evaluating new medicines for use in treating illness, it is important to determine whether they are safe to use. The genotoxic and mutagenic potential of a candidate therapeutic should be examined, since changes in genetic material can cause effects on somatic and/or germ cells and lead to the formation of cancer and/or malformation during embryonic development [14].

The present work aims to determine and compare the antiproliferative activity of β-lapachone in various neoplastic cell lines and evaluate its safety in the context of genotoxicity and mutagenicity.

## 2. Results

### 2.1. MTT Test

**Figure 1:**
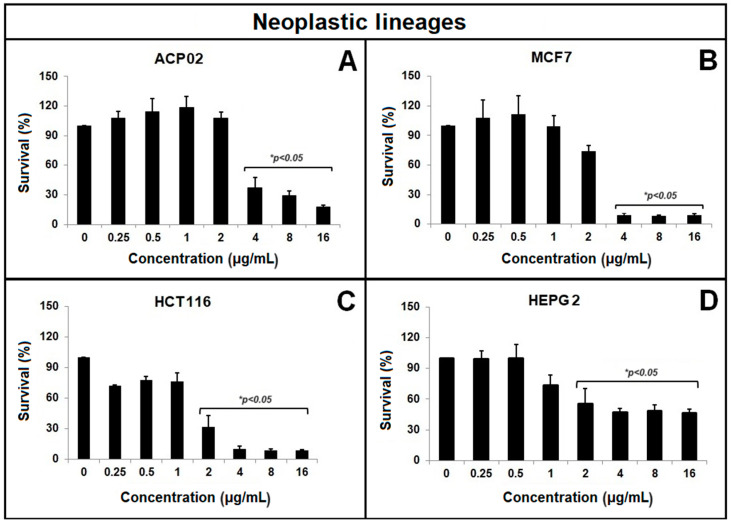
The survival of neoplastic cell lines under β-lapachone treatment was evaluated using the MTT test. The tested cell lines were (**A**) ACP02, (**B**) MCF7, (**C**) HCT116, and (**D**) HEPG2. Statistical analysis was performed using ANOVA parametric test (multiple comparisons—Tukey); * indicates a significant difference relative to the other treatments.

**Figure 2:**
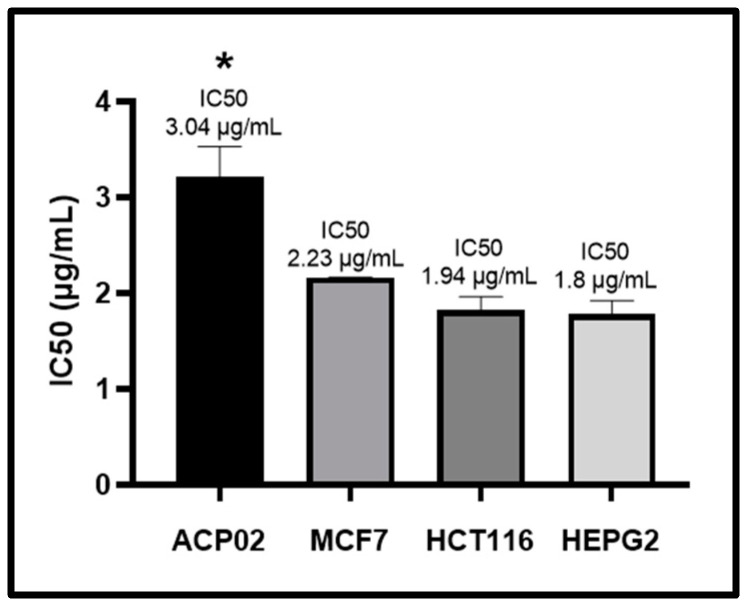
IC50 values of β-lapachone in four neoplastic cell lines. Statistical analysis was performed using ANOVA parametric test (multiple comparisons—Tukey); * indicates a significant difference relative to the other cell lines.

As shown in Figure 1, the MTT (3-(4,5-dimethylthiazol-2-yl)-2,5-diphenyl-2H-tetrazoliumbromide) assay revealed that β-lapachone dose-dependently decreased survival among four neoplastic cell lines. In the ACP02 and MCF7 cell lines, the decrease was significant from 4 µg/mL in relation to the other concentrations, while in the HCT116 and HEPG2 lines, the decrease was significant from 2 µg/mL in relation to the other concentrations. A statistical comparison of the IC50 values for each cell line (Figure 2) showed that ACP02 cells were more resistant than the other cell lines, which had IC50 values similar to one another for β-lapachone.

### 2.2. Apoptotic Index

**Figure 3:**
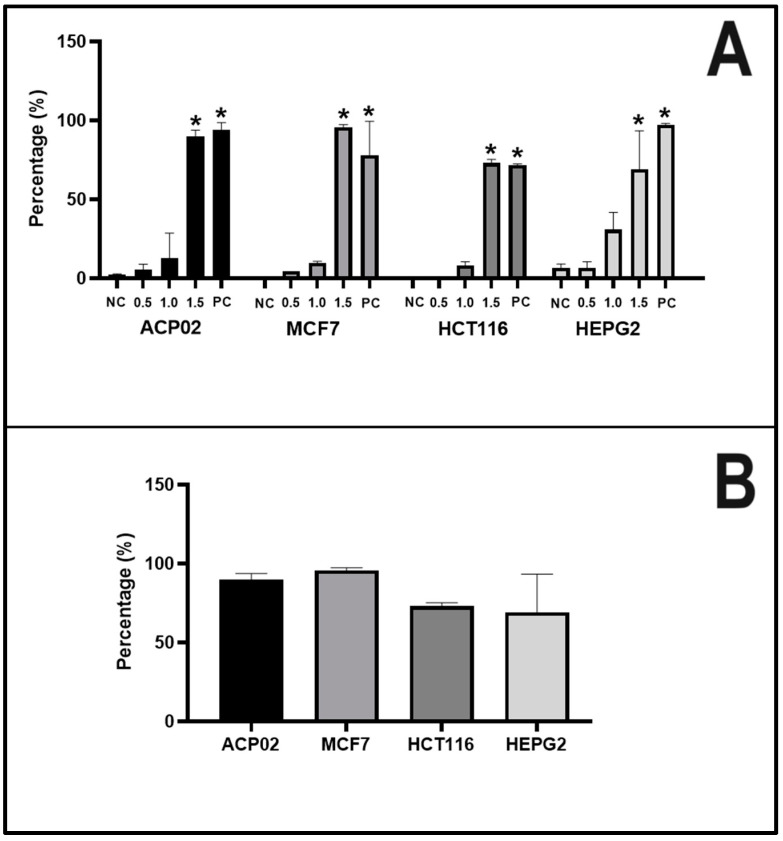
Apoptosis in neoplastic cells treated with β-lapachone for 24 h. (**A**) The cell lines were exposed to different concentrations of β-lapachone and the apoptotic index was determined. (**B**) The percentage of apoptotic cells seen at the highest concentration tested (1.5 µg/mL) was compared across the cell lines. NC: negative control; PC: positive control. Statistical analysis was performed using ANOVA parametric test (multiple comparisons—Tukey); * indicates a significant difference with respect to the NC, 0.5 µg/mL, and 1 µg/mL groups.

When we tested the apoptotic index in the four neoplastic cell lines under treatment with β-lapachone, we obtained consistent results in all four cell lines. Compared to the negative control, apoptosis was significantly induced by the highest concentration tested (1.5 µg/mL) and by doxorubicin (DOX, positive control), but not by the other tested concentrations of β-lapachone (Figure 3A). As shown in Figure 3B, we compared the percentage of apoptotic cells seen at the highest concentration tested (1.5 µg/mL) and found no significant difference among the four cell lines.

### 2.3. Comet Assay

**Figure 4:**
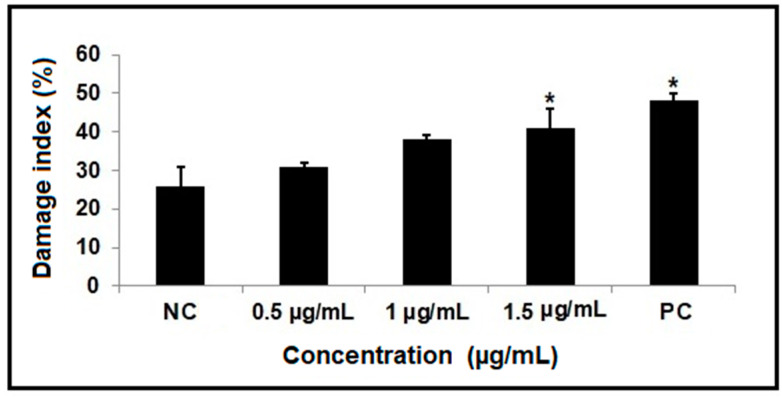
DNA damage index in HepG2 cells treated for 3 h with various doses of β-lapachone. NC: negative control; PC: positive control. Statistical analysis was performed using ANOVA parametric test (multiple comparisons—Tukey); * indicates a significant difference relative to the NC.

The comet assay was used to evaluate DNA damage. As shown in Figure 4, treatment with the highest dose of β-lapachone (1.5 µg/mL) and the positive control significantly increased the DNA damage index relative to the negative control.

### 2.4. Micronucleus Test

**Figure 5:**
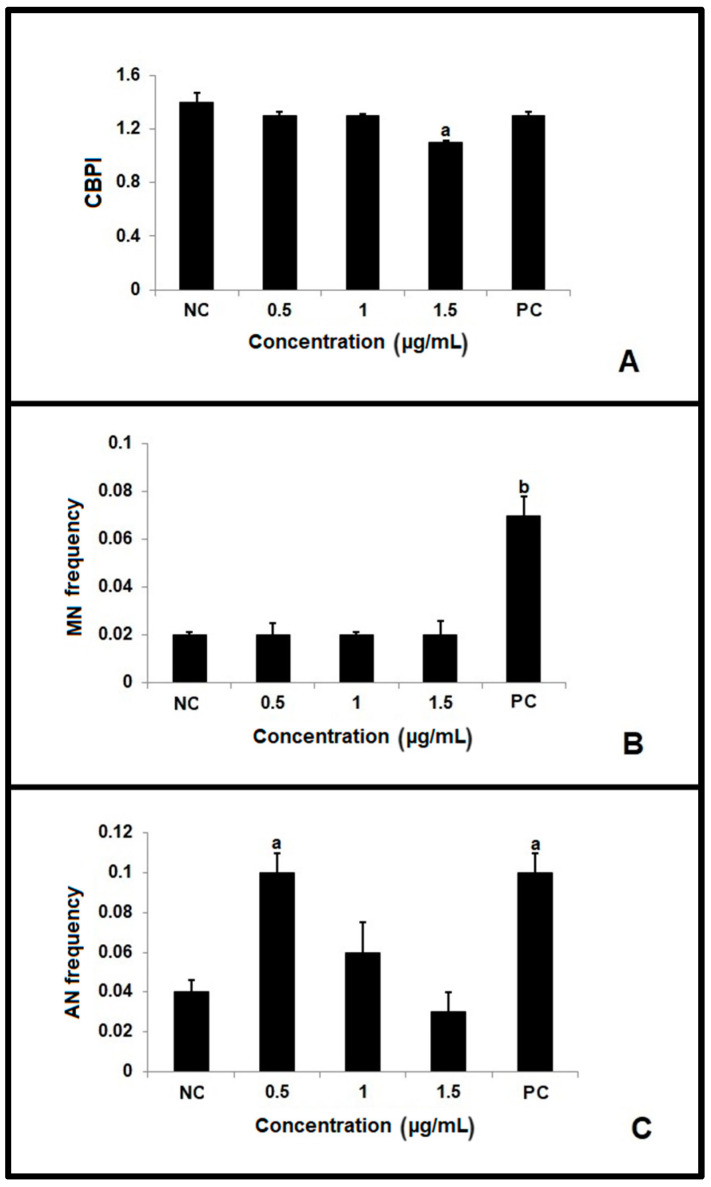
Micronucleus test results for cells exposed for 24 h to different concentrations of β-lapachone and control treatments. (**A**) Cytokinesis block proliferation index (CBPI). (**B**) Frequency of micronuclei (MN). (**C**) Frequency of nuclear abnormalities (NAs). NC: negative control; PC: positive control. Statistical analysis was performed using ANOVA parametric test (multiple comparisons—Tukey); ^a^ indicates a significant difference relative to the NC group; ^b^ indicates a significant difference relative to all other treatments.

The micronucleus test revealed that the cytokinesis block proliferation index (CBPI) values were significantly lower among cells treated with the highest concentration of β-lapachone (1.5 µg/mL), compared to the control group (Figure 5A). Regarding the MN frequency, only the positive control showed significantly higher values compared to the other groups (Figure 5B). Other nuclear abnormalities (NAs, described further below) were significantly increased in cells treated only with the lowest concentration (0.5 µg/mL) of β-lapachone and the positive control, relative to the other treatments (Figure 5C).

**Figure 6:**
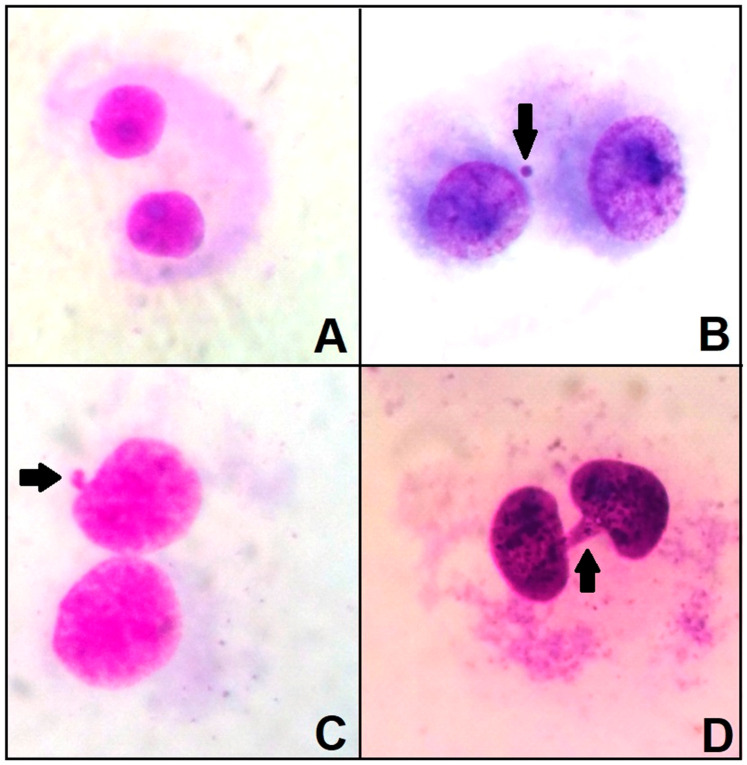
Parameters analyzed in the micronucleus test of cells exposed for 24 h to β-lapachone. (**A**) Binucleated cell without any change (negative control cells). (**B**) Binucleated cell with micronucleus (arrow) from the positive control group. (**C**) Cell treated with 0.5 µg/mL of β-lapachone, exhibiting a nuclear bud (arrow). (**D**) Cell treated with 1 µg/mL of β-lapachone, exhibiting a nucleoplasmic bridge (arrow).

Regarding the nuclear abnormalities found in treatments with β-lapachone, the presence of nuclear sprouts (Figure 6C) and nucleoplasmic bridges (Figure 6D) stood out. In the control group, the occurrence of binucleated cells without micronuclei was observed (Figure 6A). Only the positive control showed the occurrence of micronuclei in binucleated cells (Figure 6B).

## 3. Discussion

Our results are consistent with previous reports that β-lapachone exerts antiproliferative activity and is a promising candidate for treating cancer. The low IC50 found in our results shows that a very small amount of β-lapachone could cause cell death in the tested cancer cell lines. When we compared the sensitivity of the tested cell lines, we observed that ACP02 cells were relatively resistant to β-lapachone. This cell line is derived from stomach cancer; this disease is difficult to prognose and exhibits low response rates to chemotherapy or radiochemotherapy [15], and these aspects may be related to our present observations. Different cancer cell lines often vary in their sensitivity to a given agent, at least partly reflecting tumor heterogeneity and the complexity of many types of cancer [16]. However, the IC50 of β-lapachone toward ACP02 cells was around 3 µg/mL. This still represents a fairly high cytotoxic potential; previous studies reporting that β-lapachone and its derivatives had promising cytotoxicity in several human cancer cell lines based this conclusion on observing IC50 values less than 4 μg/mL [16,17].

Numerous published studies have identified various action mechanisms of β-lapachone. These include its ability to induce the formation of reactive oxygen species (ROS) via catalysis by NAD(P)H:quinone oxidoreductase 1(NQO1); this may benefit potential antitumor activity, since NQO1 is usually expressed more significantly in tumors than in normal cells [12]. Other action mechanisms include the inhibition of topoisomerase action and modification of p53 expression levels [4,18].

Regarding the involved cellular death pathway(s), different types of cancer have been associated with different cell death modes induced by β-lapachone, including apoptosis [19,20,21], autophagy [22], cytostasis [23], and necroptosis [24,25]. In this work, we observed significant β-lapachone-induced apoptosis in all four cell lines. Pro-apoptotic activity is considered advantageous in anticancer therapy, given that a cell undergoing apoptosis is phagocytized by neighboring cells or macrophages before it releases any cellular content. Necrosis, in contrast, involves the release of toxins that can spread to healthy cells in tissues near the primary tumor and thereby cause local inflammation [26,27,28,29].

Regarding the induction of apoptosis, we observed a discrepancy in the cytotoxicity values observed by the MTT test, since the apoptosis index already reveals high occurrence at doses below the IC50 detected by the MTT. These data corroborate a previous report that the MTT test has lower sensitivity for detection [30]. The authors suggested that the fluorescent dye used to detect apoptosis increases the sensitivity of the test, as it allows visualization of morphological changes characteristic of early-stage apoptosis, prior to the decrease in mitochondrial respiration that is detected by the MTT test. Regardless, our results obtained with both assays indicated that β-lapachone has high cytotoxic potential in the four cancer cell lines tested herein.

Despite the previous and present findings, many challenges remain unresolved regarding the potential use of β-lapachone in anticancer therapy, including issues related to its solubility and bioavailability in the body [4,18]. Various strategies have been used in an attempt to overcome these issues. For example, β-lapachone-containing nanotherapeutics have been developed and shown to exhibit greater antiproliferative effects than free β-lapachone, along with the ability to target the cancer site and/or cancer cells [31,32,33]. Other efforts have sought to identify β-lapachone derivatives with improved biological potency, solubility, and/or bioavailability [18,34,35].

Further work is still needed to fully assess the safety profile of β-lapachone, including any potential secondary effects on healthy cells and/or at sublethal concentrations. The present work is one of the few studies that has evaluated the potential for β-lapachone to cause genotoxicity and mutagenicity. The nuclear abnormalities observed in the micronucleus test may support a previous suggestion that the formation of nucleoplasmic bridges and that of nuclear buds are connected via induction of the break–fusion–bridge cycle [36]. In this model, a genotoxic substance causes breaks at the ends of chromosomes, leading them to attach to their sister chromatids. The pairs thus remain together during the anaphase and when chromosomes seek to migrate to opposite poles, forming the nucleoplasmic bridge. An attached chromosome will tend to break unevenly, generating gene amplifications in some cells. In this model, the amplifications may tend to localize to the periphery of nuclei, where they form nuclear buds.

Notably, we observed more frequent nuclear abnormalities at the lower concentrations tested. We speculate that the high level of DNA damage caused by the highest concentrations of β-lapachone (detected by the comet assay) could trigger DNA-damage-related cell death, meaning that nuclear abnormalities were seen less frequently. This fact could be confirmed by the increase in apoptosis at the highest concentration, a factor that may have been induced by the occurrence of excessive DNA damage [18,37,38]. In this sense, the β-lapachone-induced increase in DNA damage detected in the comet assay appears to be directly related to the ability of this agent to induce NQO1-catalyzed ROS generation [18]. Another factor that may be involved in the ability of β-lapachone to induce nuclear abnormalities only at lower concentrations is the possible cytostatic effect of higher concentrations, as evidenced by our CBPI results. In this case, the changes would not be visualized through the micronucleus test because the cells do not progress through the cell cycle to complete mitosis. A cytostatic effect of β-lapachone has also been reported by other authors [4,16,39].

In sum, the nuclear changes herein detected in cancer cells treated with relatively low concentrations of β-lapachone should be considered further as potential side effects relevant to the potential use of β-lapachone in anticancer therapy.

## 4. Materials and Methods

### 4.1. Sample Collection and Extraction and Characterization of β-Lapachone

Sample collection and extraction and characterization of β-lapachone were carried out by the research group at the Laboratory of Pharmacology and Neglected Diseases at the Federal University of Pará (UFPA). All β-lapachone used in this work was extracted from sawdust of Ipê wood, which was collected from a sawmill located in the state of Pará, Brazil.

For extraction of β-lapachone, 1.859 g of sawdust was mixed with 10 L of 1% NaHCO_3_ solution. The mixture was left to rest for 45 min and then filtered. The pH of the extract was adjusted to pH 3 with 6M HCl until the red solution changed color, forming a yellow precipitate. The material was filtered and was retained and placed in a desiccator to remove moisture. To obtain lapachol, the residue was scraped off the filter paper and 5.540 g of this material was fractionated on a chromatographic column (125 g of MERK silica gel 70–230 mm; height, 38 cm; and width, 4 cm), using dichloromethane as the mobile phase. Thin layer chromatography (TLC) was performed using dichloromethane–hexane (8:2) as the eluent. The fraction richest in lapachol was fractionated again and the fraction with the highest pure lapachol content was chosen for use.

The structural determination of lapachol was carried out using nuclear magnetic resonance spectroscopy (NMR) of hydrogen (1H NMR) and carbon 13 (13C NMR). The 1H NMR and 13C NMR spectra were obtained by a Varian Unity Plus 300 apparatus using deuterated chloroform (CDCl3) and tetramethylsilane (TMS) solution as internal references. The chemical shift values were measured in parts per million (ppm) in relation to the TMS and the coupling constants (J) in Hertz (Hz).

To synthesize β-lapachone, 500 mg of lapachol was mixed with concentrated sulfuric acid (1.5 mL) in an ice bath for approximately 10 min and then ice-cold distilled water (50 mL) was added. This solution was filtered and recrystallized with ethanol and the obtained orange solid was solubilized in deuterated chloroform. Nuclear magnetic resonance analysis (Bruker Advance DPX 200 NMR Spectrometer, Billerica, MA, USA) was used to confirm the structure of β-lapachone (Supplementary Materials; Figures S1 and S2, Table S1).

### 4.2. Cell Lines and Growing Conditions

For in vitro tests, the following human cells of different neoplastic tissue origins were used: ACP02 (gastric adenocarcinoma cells), MCF7 (breast carcinoma cells), HCT116 (colon cancer cells) and HEPG2 (hepatocellular carcinoma cells). Some cells were purchased from the Rio de Janeiro Cell Bank and others were kindly provided by the Human Cytogenetics Laboratory at UFPA.

All cell lines were cultivated in DMEM (Dulbecco’s Modified Minimum Essential Media) supplemented with 10% Fetal Bovine Serum (FBS) and amphotericin (2.5 μg/mL). The cells were kept in plastic bottles and incubated in a sterile incubator in an atmosphere of 5% CO_2_ at 37 °C until the tests were carried out.

### 4.3. Cell Viability

#### 4.3.1. MTT Test

Cells were seeded in a 96-well plate at 5 × 10^4^ per well and the plates were incubated in a CO_2_ incubator at 37 °C. After 24 h, the wells were loaded with seven concentrations of β-lapachone (0.25–16 µg/mL) that were chosen based on our preliminary tests and previous reports in the literature. DMEM was used as a negative control and doxorubicin (DOX, 100 µg/mL) was used as a positive control. After 24 h of exposure, the cells were exposed to 100 μL of MTT (5 mg/mL) for 3 h. The MTT was removed and 100 μL of DMSO was added to each well. After 1 h, the absorbance was read on a spectrophotometer (Epoch Biotek, Winooski, USA) at a wavelength of 570 nm using the Gen5 program (2003) version 2.03.1. Cell viability (%S) was determined using the following formula: %S = 100 × [(Atested − Ablank)/(Acontrol − Ablank)], where A represents the absorbance of each well and blank represents the results from an empty well. The IC50 (inhibitory concentration of 50% of cells) was calculated using the Prisma GraphPad version 5 program.

#### 4.3.2. Apoptotic Index

Cells were seeded in a 12-well plate at 8 × 10^4^ cells/well and incubated in a CO_2_ incubator at 37 °C containing 95% air and 5% CO_2_. After 24 h, the cells were exposed to 0.5 µg/mL, 1 µg/mL, and 1.5 µg/mL of β-lapachone (values corresponding to sublethal doses: approximately 25, 50, and 75% of the IC50). DMEM was used as the negative control and DOX (100 µg/mL) was used as the positive control. After 24 h of exposure, the cells were trypsinized and removed from the plate. Cells were centrifuged in a Falcon tube; 50 μL of cells were mixed and removed and 2 μL of acridine orange solution (100 µg/mL) was used to prepare the slides. Analysis was carried out using a Nikon fluorescence microscope (HS 50S) with a 40× objective and FITC filter. Three hundred cells were counted per treatment, based on previous reports [40,41], and the analytic parameters were as previously described [42]. Cells were defined as follows: viable cells were stained green and had organized chromatin and apoptotic cells were stained green and had highly fragmented chromatin (presence of apoptotic bodies). The percentage of apoptotic cells was calculated as follows: % of apoptotic cells = 100 × (number of cells in apoptosis/total cells).

### 4.4. Genotoxicity Assays

For the genotoxicity assays, we used HepG2 cells. This cell line is commonly recommended for such work because its hepatic origin and capacity to metabolize compounds improve its ability to reflect possible correlations with in vivo effects [43]. HepG2 cells were seeded in 12-well plates at 8 × 10^4^ cells/well, maintained for 24 h in a sterile incubator at an atmosphere of 5% CO_2_ and 37 °C, and then exposed to 0.5 µg/mL, 1 µg/mL, and 1.5 µg/mL of β-lapachone (approximately 25, 50, and 75% of the IC50 determined by the MTT test, respectively). DMEM was used as the negative control and 5 µg/mL of DOX and 0.02 µg/mL were used as positive controls for the comet assay and micronucleus test, respectively. Treated cells were trypsinized and the comet assay and micronucleus test were performed.

#### 4.4.1. Comet Assay

The comet assay was carried out according to a modified version of the published methodology [44]. After 3 h of exposure to β-lapachone, the cells were collected and resuspended in DMEM, and 20 µL of cell suspension was mixed with 0.5% low-melting-temperature agarose. The mixture was placed on slides that had been pre-coated with normal agarose (1.5%) and covered with a coverslip. The slides were incubated for 15 min at 4 °C and then placed in a lysis solution (2.5 M NaCl, 100 mM EDTA, and 10 mM Tris; pH 10.0–10.5) containing 1% Triton X-100 and 10% DMSO. After 24 h, the slides were transferred to a horizontal electrophoresis vat and covered with alkaline buffer (300 mM NaOH and 1 mM EDTA, pH > 13) for 30 min and then subjected to electrophoresis for another 30 min at 0.8 V/cm. Finally, the slides were washed three times for 5 min each with deionized water and then fixed for 5 min in P.A. ethyl alcohol. The slides were dried and stored in a refrigerator until analysis. The slides were stained with ethidium bromide (20 μg/mL) and analyzed using a Nikon H550S fluorescence microscope (510–560 nm filter, 590 nm filter barrier, and 40× magnification). Comets were categorized based on tail size [45] and 100 cells were analyzed per treatment. The damage index (DI) was calculated as a percentage using the following formula: DI = 100 × [(1 × n1) + (2 × n2) + (3 × n3) + (4 × n4)/n], where n represents the total number of cells analyzed and n1 to n4 represent the number of cells with damage levels from 0 to 4, respectively.

#### 4.4.2. Micronucleus Test

The micronucleus test was carried out according to the standard protocol [46], with Cytochalasin B (6 µg/mL) added 24 h before the cells were removed. After 24 h of exposure to β-lapachone, the cells were trypsinized, transferred to a Falcon tube, treated with 2.5 mL of hypotonic solution (KCl) for 3 min, and then treated with 2 mL of Carnoy’s fixative (methanol–acetic acid, 3:1). The cells were centrifuged, the supernatant was removed, another 1 mL of Carnoy’s fixative was added, and the sample was stored at 4 °C. For analysis, the cells were dripped onto slides and analyzed under a Zeiss optical microscope with a 40× objective. For each slide, 500 cells were analyzed and quantified. The cytokinesis block proliferation index (CBPI) was calculated using the following formula: CBPI = [M1 + 2(M2) + 3(M3) + 4(M4)]/N, where M1, M2, M3, and M4 represent the number of cells with 1, 2, 3, and 4 nuclei, respectively, and N is the total number of viable cells. To calculate MN and other nuclear abnormalities (NAs), 1000 binucleated cells per slide were assessed for micronuclei or other NAs, and their frequencies were calculated as follows: fMN or NA = nºMN or NA/1000.

### 4.5. Statistical Analysis

Statistical analyses were performed using an ANOVA or Kruskal–Wallis test, which were chosen according to the normality standard of the data defined using the Kolmogorov–Smirnov test. All statistical analyses were carried out using the Bioestat 5.0 program (*p* ≤ 0.05).

## 5. Conclusions

Our present results and the previous reports clearly support the idea that β-lapachone may be a promising candidate as a cancer chemotherapeutic. However, it will be important to further evaluate its safety for use, especially at sublethal concentrations. Here, we have shown that β-lapachone induces genotoxic effects in vitro at relatively low exposure concentrations. Therefore, further studies are needed to establish threshold exposure concentrations. Moreover, additional studies should be carried out using technologies and/or β-lapachone analogs for targeted delivery to the relevant organ(s), thereby improving the therapeutic benefits and minimizing adverse effects.

## Data Availability

Data are available from the corresponding author upon request.

## Supplementary Materials

The following supporting information can be downloaded at https://www.mdpi.com/article/10.3390/molecules29061395/s1: Figure S1: Representation of the hydrogen (H1) nuclear magnetic resonance spectrum of β-lapachone (CDCl3, 200 MHz); Table S1: Data from the attributions of nuclear magnetic resonance signals from β-lapachone (1H 200 MHz,13C 50 MHz, CD3OD); Figure S2. Representation of the carbon 13 (C13) nuclear magnetic resonance spectrum of β-lapachone (CDCl3, 200 MHz).
