# Supplementary material for: Anticancer Potential and Safety Profile of β-Lapachone In Vitro
Source: Molecules. 2024 Mar 21;29(6):1395. doi: 10.3390/molecules29061395 (PMC10975997; doi:10.3390/molecules29061395)
Supplement: Supplementary file 1 [file molecules-29-01395-s001.zip › molecules-2795492-supplementary.pdf]

Supplementary Material

Characterization of β-lapachone

The orange crystals obtained from the acid synthesis of lapachol were subjected to 1H NMR analysis, observing characteristic signs of β-lapachone: singlet 1.47 with integral for 6 hydrogens, referring to methyl (3H-1' and 3H- two'). Two triplets in δ1.8 and δ 2.5, with an integral for two hydrogens referring to the methylene hydrogens H-4, and the second triplet is the hydrogens of H-3, which are close to the oxygen of the heterocyclic ring, which makes them become more unprotected. Two double doublets, at δ 8.0 corresponding to H-9, become more unshielded due to their proximity to the carbonyl, and at δ 7.8 attributed to H-12 while H-10 and H-11 correspond to both double triplets at δ 7.5 and δ 7.6 refer to the aromatic ring (**Figure S1**; **Table S1**). In relation to the NMR spectrum obtained from Carbon (C), the following signals were obtained: δ 31.6 (C-4); δ 162.0 (C-1); δ 112.7 (C-2); δ 178.6 (C-6); δ 130.1 (C-13); δ 132.6 (C-8); δ 179.9.0 (C-7); δ 134.8 (C-9); δ 132.6 (C-5); δ 16.1 (C-3); δ 124.6 C-12); δ 26.7 (C-1′ and C-2′) (**Figure S2**; **Table S1**).

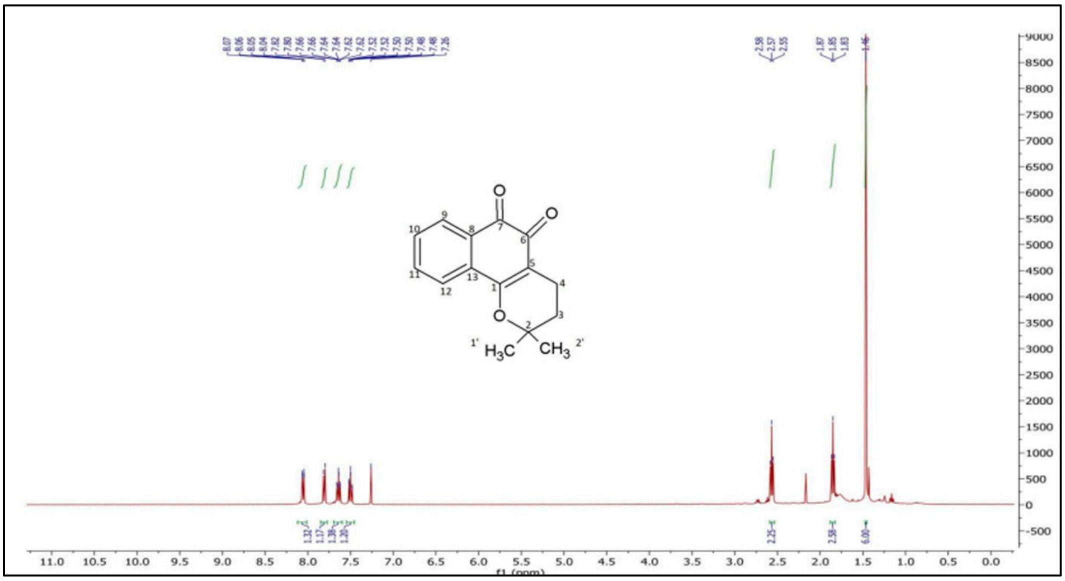

Figure S1. Representation of the hydrogen (H1) nuclear magnetic resonance spectrum of β-lapachone (CDCl3, 200 MHz).

Table S1. Data from the attributions of nuclear magnetic resonance signals from β-lapachone (1H 200 MHz,13C 50 MHz, CD3OD).

Position	δ ^{1}H (ppm)	δ ^{13}C (ppm)
1	-	162,0
2	-	112,7
3	1,87 t	16,1
4	2,58 t	31,6
5	-	132,6
6	-	178,6
7	-	179,9
8	-	132,6
9	8,-7 dd	134,8
10	7,62 td	128,6
11	7,66 td	130,6
12	7,82 dd	124,6
13	-	130,1
1’	1,46 s	26,7
2’	1,46 s	26,7

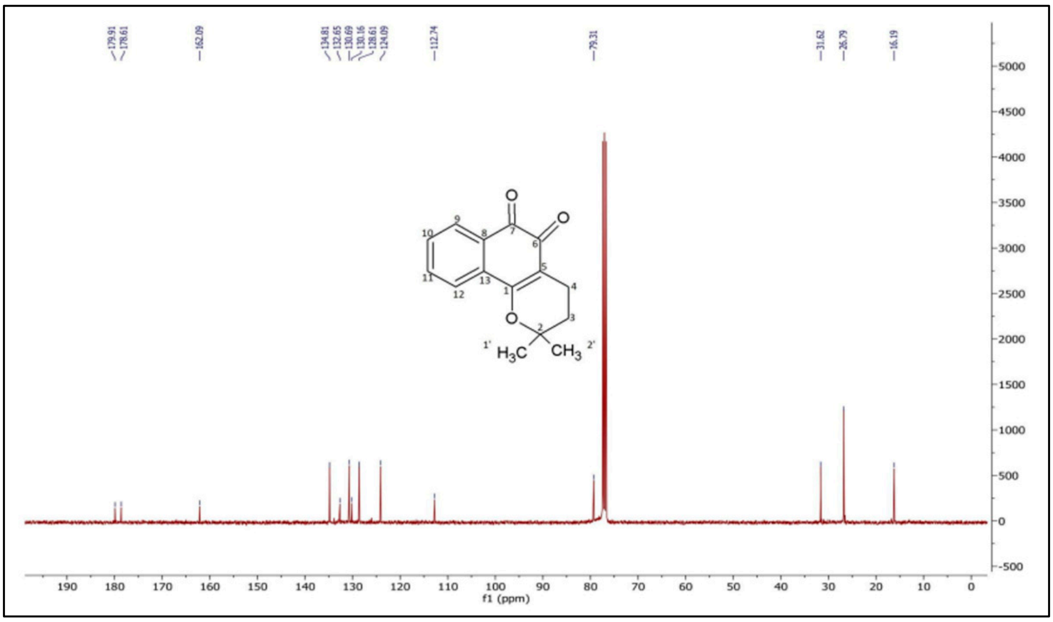

Figure S2. Representation of the carbon 13 (C13) nuclear magnetic resonance spectrum of β-lapachone (CDCl3, 200 MHz).
